# An innovative approach in monitoring oral cholera vaccination campaign: integration of a between-round survey

**DOI:** 10.1186/s12889-022-12610-5

**Published:** 2022-02-05

**Authors:** Jerôme Ateudjieu, Martin Ndinakie Yakum, André Pascal Goura, Maureen Tembei Ayok, Etienne Guenou, Corine Blondo Kangmo Sielinou, Frank Forex Kiadjieu, Marcellin Tsafack, Ingrid Marcelle Douanla Koutio, Ketina Hirma Tchio-Nighie, Hervé Tchokomeni, Paul Nyibio Ntsekendio, David A. Sack

**Affiliations:** 1 Department of Health Research, M.A. SANTE (Meilleur Accès aux soins de Santé), Yaounde, Cameroon; 2 Department of Public Health, Faculty of Medicine and Pharmaceutical Sciences, University of Dschang, Dschang, Cameroon; 3 Division of Health Operations Research, Ministry of Public Health, Yaoundé, Cameroon; 4 Faculty of Sciences, University of Buea, Buea, Cameroon; 5 Johns Hopkins Bloomberg School of Public Health, Baltimore, USA

**Keywords:** Monitoring and evaluation, OCV, Cholera, Survey, Campaign - Mogode - Far North, Cameroon-Vaccination coverage -immunization

## Abstract

**Background:**

Monitoring and Evaluation (M&E) is essential in ensuring population’s access to immunization. Surveys are part of this M&E approach but its timing limits the use of its results to improve the coverage of the evaluated campaign. An oral cholera vaccination campaign was organized in a health district of the Far North region of Cameroon and involved an innovative M&E approach. The aim of this project was to assess the feasibility and effect of using recommendations of a community-based immunization and communication coverage survey conducted after the first round of an OCV campaign on the coverage of the second-round of the campaign.

**Methods:**

Two community-based surveys were included in the M&E plan and conducted at the end of each of the campaign rounds. Data were collected by trained and closely supervised surveyors and reported using smartphones. Key results of the first-round survey were disseminated to campaign implementing team prior to the second round. The two rounds of the pre-emptive campaign were organized by the Cameroon Ministry of Public Health and partners with a two-week interval in the Mogode Health District of the Far North region of Cameroon in May and June 2017.

**Results:**

Of 120 targeted clusters, 119 (99.1%) and 117 (97.5%) were reached for the first and second rounds respectively. Among the Mogode population eligible for vaccination, the immunization coverage based on evidence (card or finger mark) were estimated at 81.0% in the first round and increased to 88.8% in the second round (X^2^=69.0 and p <0.00). For the second round, we estimated 80.1% and 4.3% of persons who were administered 2 doses and 1 dose of OCV with evidence respectively, and 3.8% of persons who have not been vaccinated. The distribution of campaign communication coverage per health area was shared with the campaign coordination team for better planning of the second round campaign activities.

**Conclusions:**

It is feasible to plan and implement coverage survey after first round OCV campaign and use its results for the better planning of the second round. For the present study, this is associated to the improvement of OCV coverage in the second-round vaccination. If this is persistent in other contexts, it may apply to improve coverage of any health campaign that is organized in more than one round.

**Supplementary Information:**

The online version contains supplementary material available at 10.1186/s12889-022-12610-5.

## Background

Cholera is still a major public health problem worldwide with 1.3 million to 4.0 million cases and 21 000 to 143 000 deaths yearly [1]. The greatest burden of the disease is borne by resource-limited countries recording the highest attack rate as well as case fatality at yearly basis [1]. Cameroon is among the frequently affected countries with sporadic outbreaks associated with high attack and case-fatality rates [2]. During the ten previous years, Cameroon has recorded several cholera outbreaks in which the outbreak of 2009-2012 was the most serious in terms of the number of regions/districts affected, number of cases reported and high case fatality rate [2, 3]. As recommended by the Global Task Force for Cholera Control and WHO, Oral Cholera Vaccination campaign is one of the main strategies to reduce the burden of the disease [4]. As recommended from the National cholera Contingency plan, a need assessment conducted by actors involved in cholera control in Cameroon in 2015 identified Mogode health district as most vulnerable for cholera and top to be prioritized for cholera vaccination campaigns.

Leading organizations working for cholera control have provided recommendations on how to use the vaccine [5, 6]. It comes out from these recommendations that OCV campaign is needed to respond or prevent outbreaks and that two doses are needed to induce expected immunization. During vaccination campaigns, many studies have reported high coverage in the first round and low coverage in the second round, with a gap in some settings of up to 25% [7–10]. From published data, there is no evidence documenting interventions that have significantly improve the dropout rate between the first and second rounds [11–13]. The performances in terms of coverage of vaccination campaigns planned in more than one-round may be limited by a dropout rate from the first round to the next, but it also offers the opportunity to correct the shortcomings of the first round of immunization in subsequent rounds. The challenge is to carry out corrective activities within time separating the vaccination rounds. OCV immunization campaigns are organized in two rounds in two-week interval as recommended by the manufacturers.

Monitoring and Evaluation (M&E) is recommended to be part of OCV campaign the same way it is for all other campaigns in order to ensure the appropriate implementation of planned activities [14]. This activity helps to track and report information about a project or program’s activities with the aim of improving its outcome and impact [15]. From current practices in Cameroon, M&E is part of most immunization campaigns but is mostly limited to in-process and end-process monitoring and results are shared after the campaign; thus, cannot be used to improve the evaluated campaign. Such limitations have been documented in similar health interventions [13, 16, 17]. Since the success of a campaign is determined by the quality of planning, preparation, implementation and evaluation; current M&E must be integrated in all of these campaign phases to generate timely information for identification of weaknesses associated with each of these steps.

Part of the resources allocated to M&E are devoted to end-campaign survey but the results and recommendations of these surveys are shared much later and cannot be used to improve the coverage of the said campaign. OCV campaign as many other campaigns are organized in two rounds and fails to include survey as M&E activities to identify weaknesses regarding the coverage and quality of campaign interventions from the first round so as to improve the second round [18, 19]. Whereas the mapping of areas and populations not reached by the first round can guide the adjustment of communication and vaccination strategies for the second round.

An OCV immunization campaign was organized in Mogode health district during May-June 2017 by the Cameroon Ministry of Public Health and partners. This campaign targeted 126,619 people, one year and above (excluding pregnant women) with vaccine administered in two rounds using door-to-door as main strategy. M.A. SANTE (*Meilleur Accès aux soins de Santé*) a Cameroon-based NGO, was in charge of M&E, and adapted a new approach which was inclusive of all the other traditional activities but extended to a first round post campaign survey. The survey and campaign M&E results were shared to the campaign team to plan the second round organized two weeks after the first round and implement corrective decisions. The M&E activity was part of the Cameroon Ministry of Health OCV campaign application that was approved by OCV working group of the Global Task Force on Cholera Control. This project was conducted with the objectives to determine whether (1) the presentation of recommendations of a community-based immunization and communication coverage survey conducted after the first round to partners involved in the two-round OCV campaign can contribute to improving the coverage of the second-round campaign and thus, the whole campaign? And whether (2) the organization of training for actors involved, before the campaign, the on-site monitoring of data collection, online data transmission, rapid data processing and analysis is feasible within two weeks and can allow the dissemination of results of a survey conducted at the end of the first-round campaign before the second round planned two weeks after the first?

## Methods

### Design

At the end of the first vaccination round, a five-day community-based survey was conducted at household level to estimate the vaccination and communication coverage, and reasons for non-vaccination among targeted population. Data were collected using Open Data Kit (ODK) forms in Smartphone, by eight teams of three surveyors, reviewed, validated and submitted online daily by the supervisor of each team. The feasibility of the proposed innovation was assessed by determining whether the sequence and timing of activities contributing to the implementation of the post-first round survey allowed the recommendations of this survey to benefit the second round of vaccination. This benefit was assessed by comparing the immunization coverage estimated by the survey conducted after each round of the OCV campaign.

### Setting

**Fig. 1:**
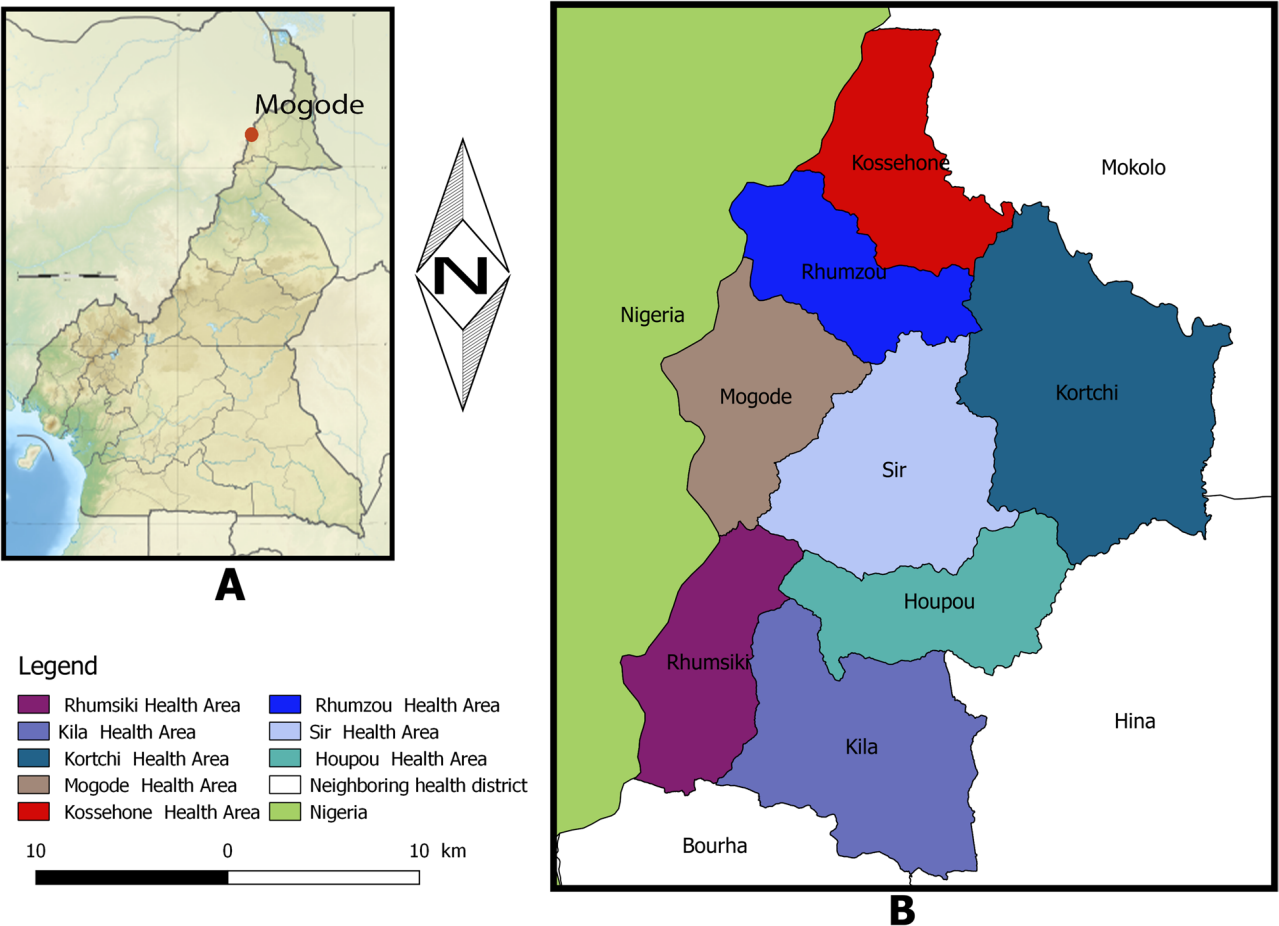
Mogode OCV: Map of Cameroon in Africa showing study sites in Mogode Health District in the Far North region (The map was produced using the software Qgis 2.18; the figure is owned by authors)

Mogode health district (MHD) is one of the 30 health districts of the Far North region of Cameroon, composed of eight [8] health areas and 14 healthcare facilities. It is a rural and impoverished health district that lacks basic services like access to water, road, and electricity. Water is not supplied by the national water distribution company and the population relies on wells and bore-holes for water supply. The geographical characteristics do not permit the population to construct wells as a water source like it is in many Cameroonian localities. It shares boundaries with the Adamawa state of Nigeria and 3 health districts in Cameroon including Hina, Mokolo, and Bourrha. It is a point of very high population exchange between Nigeria and Cameroon with many refugees fleeing from Boko Haram insurgency and crossing to go to the Menawao refugee camp. Its health facilities receive patients from both Cameroon and Nigeria. The 2014 cholera outbreak started in Mogode and an investigation found that the first case came from Nigeria to seek care in the Mogode district hospital. Figure 1 shows the map of Mogode and neighboring districts.

Figure 1 shows Location of the Mogode health district in the map of Cameroon (A) and health areas in the Mogode health district (B).

### Tools development

Two data collection tools were developed, one to assess the immunization coverage and reasons for non-vaccination among targeted population and the other to assess the communication coverage among heads of households. The two questionnaires were adapted from existing guide [20]. The key variables of the first questionnaire included age, gender, vaccination status, date of vaccination, evidence of the vaccination (booklet and finger-paint) and reasons of non-vaccination. The key variables of the second questionnaire included the age, gender, vaccination awareness and awareness channels for head of households. Pre-tested and validated data forms were adapted and deployed using Open Data Kit (ODK) in smartphones. Standard data collection procedures were developed for surveyors as well as guidelines for questionnaire validation used by field supervisors.

### Sample size

#### For each survey round

To estimate the required sample size for the surveys, we assume a vaccination coverage of 50% because of lack of previous information since it will give the minimum sample size [21]; a non-response rate of 20% since we were dealing with a list of households recently used for mosquito net distribution as our sampling frame, we expected that some selected households might not be found or have been transferred or refuse to participate; a precision of 7%, design effect of 2 and 95% confidence interval. Based on these, the minimum sample size for the survey was 490 persons. However, we planned to analyze data in different population subgroups. These included male and female (10 years and above), and children (aged 1-9 years).To make sure that we have enough number of participants for each of the population subgroups, we multiplied the minimum sample size by 3, giving a total of 1470 persons. For feasibility reasons, we decided to select a total of 120 clusters which means approximately 13 households per cluster.

#### For the comparison of vaccination coverage between the first and second round surveys

There is need to enroll 2560 participants per survey to compare documented vaccination coverage between the first and second round surveys assuming identical size in the two surveys, type 1 error α = 5%, power=90%, a 50% coverage in the two surveys and a precision between 5% and 10%, and a non-response rate at 20% [22].

### Participant’s selection

#### Household

Prior to the campaign, there was an insecticide impregnated mosquito net distribution campaign in December 2016 and all households in the district were enumerated in a database. This list was obtained from the district head and used as our sampling frame. In the list, neighboring quarters and households followed each other. In this way, the selected households in each cluster were neighbors. Clusters of 13 households each were formed from the sampling frame using the household unique identifiers in which a total of 1680 clusters were formed. Therefore, 120 clusters were selected using systematic sampling with a sampling interval of 14. The number of clusters assigned per health area was proportional to the total population of each health area. It is worth noting that cluster selection during each round was completely independent. In each selected cluster, all the households were included in the assessment.

#### Participants

For the purposes of assessing vaccination coverage, all individuals living in the selected households aged above 1 year and not pregnant (counter indications to vaccination) were included in the assessment. However, only the head of household or his representative was interviewed on the communication coverage. Households who refused to participate were excluded.

### Data collection

Data collection was done by 8 trained teams each composed of three surveyors and one supervisor. In each cluster, households were identified with the help of local residents and using the household’s unique identifier together with the information of the head of household. The questionnaire on immunization coverage was administered individually to each eligible person but could be responded by the guardian or any adult at home for children and elderly people (Additional file 1). Complementary data were collected by reviewing the OCV card if it existed and by checking the left index finger of the participant for vaccination mark. For children whose exact ages could not be given, this was verified from any official document or tracked using key events (local calendar) surrounding his/her birth period. On the other hand, one questionnaire on communication coverage was administered to the head of household or his representative.

All data collection was done in Open Data Kits (ODK) collect installed in smartphones and transmitted to the central server using internet after cross-checking in the field by the supervisors.

A household was considered closed when declared by an immediate neighbor or absent after three consecutive visits within the data collection period. Closed or non-consenting households were not replaced. If the inhabitants were temporarily absent, the survey team had to visit up to three times on three different days during the survey week for the household to be considered closed. Similarly, adults who were not available for the survey after three visits to the household on three different days were considered absent.

Surveyors were recruited from the community of Mogode where the campaign was implemented as they had to be able to read and write in English or French and speak in local language. The phones were android and dedicated solely to the survey. The survey teams were provided with power banks to anticipate battery drain during the survey and backup phones to replace broken ones. These phones were collected at the end of the day and returned each morning at the beginning of the survey.

### Data management

Data quality control was done at two levels. The first level was done in the field by the supervisors before transmitting data to the central server and the second level was controlled by the data manager monitoring the data in server. From the central server, the database was downloaded and cleaned on daily basis. Coherence, consistency and completeness verification was done by interacting with each field team. At the end of each survey, pre-validated key variables were rapidly analyzed by the team in charge and used to prepare the presentation to be shared with the campaign coordination team.

### Data analysis

Data analysis included analyzing survey data per and between campaign rounds. Since our sampling approach was based on clusters, we performed weighted analysis to account for inter-cluster heterogeneity. For each survey, we estimated the survey coverage and the response rate per health area, and sub group with 95% confidence interval. Communication coverage was estimated during the first survey only. Regarding communication, we estimated the proportion of households aware of campaign interventions and its distribution per health area. For vaccination coverage, we estimated per health area, sub group and overall, the proportion of participants vaccinated with evidence (vaccination card for the two rounds and finger paint for the second round only), the proportion of participants vaccinated based on recall, reasons for non-vaccination and zero-dose with 95% confidence interval. For the second-round survey, we estimated the proportion of persons who received two doses and one-dose as well. We equally compared between the two surveys the proportions of participants who received one vaccination dose with evidence and based on recall using the Pearson Chi-square test with *p*-value < 0.05 considered as significant using Epi info 7.2.2.6 software.

### Innovative intervention

**Fig. 2:**
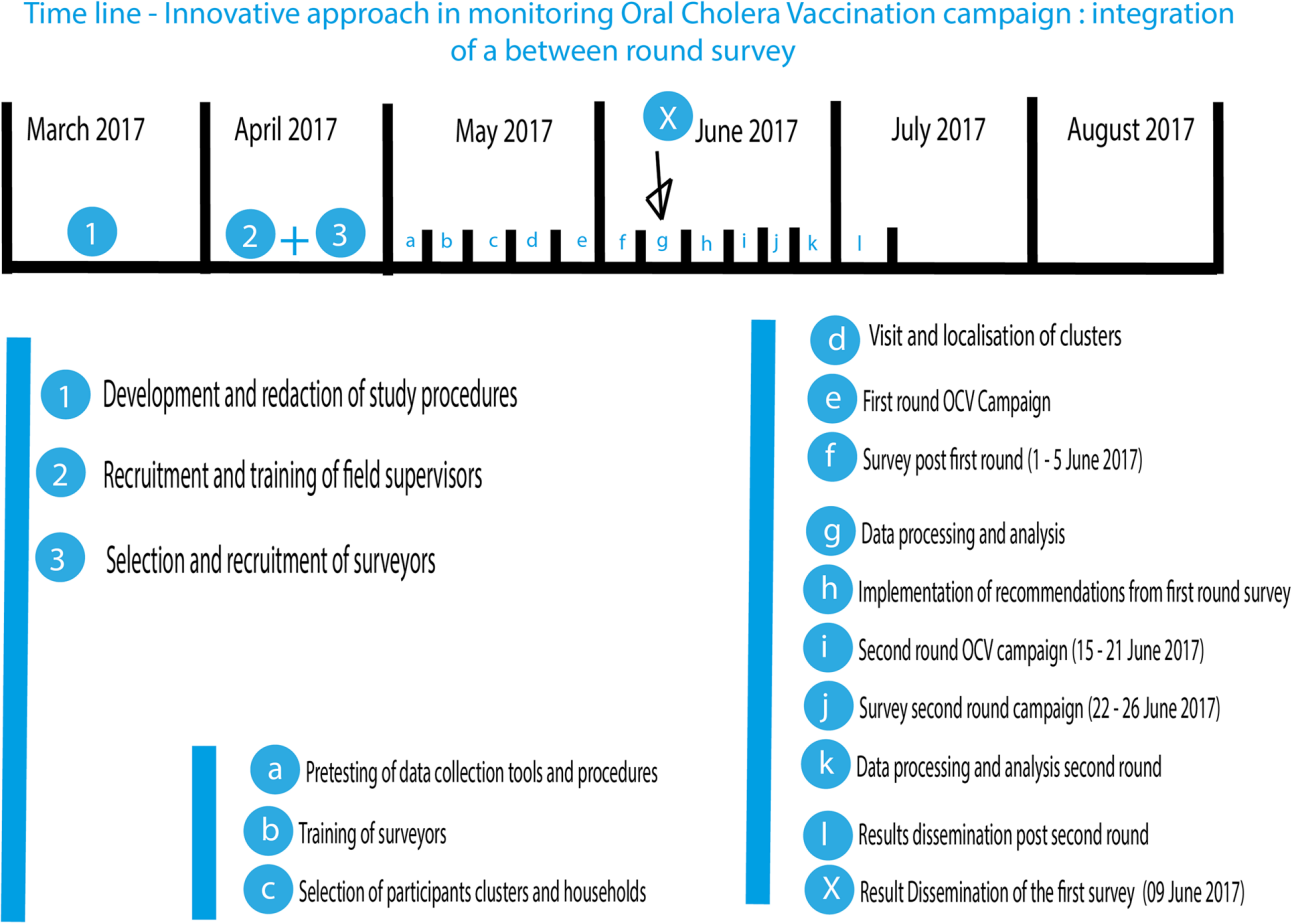
Timeline of the implementation of activities, result dissemination and surveys conducted after first and second rounds OCV campaigns

We innovated by conducting a survey at the end of the first round, timely analyzing the collected data and sharing its results with involved partners to induce corrective actions, to improve observed weaknesses during the second round planned 14 days later. A second survey was conducted at the end of the second round to compare coverages of the first and the second rounds and to estimate the benefit between rounds. Expected challenges included the limited time to collect, transmit, clean, analyze data, sharing its result and implementing its recommendations in 14 days. We addressed these challenges by (i) designing a rigorous timeline for developing and pre-testing data collection instruments; training of supervisors, monitors and surveyors, data collection, cleaning, analyzing, results dissemination, discussion and prioritization of recommendations. The training of supervisors was done before the data collection process. (ii) assigning a data collection monitor for each field team, (iii) a supervisor to supervise field activities on daily basis, (iv) conducting a day-to-day data cleaning of the database per variable, participant and cluster, (v) the date for results dissemination was scheduled with the involved partners and they were prepared to implement the resulting recommendations. The first-round campaign was implemented from the 25th to 31st May 2017. The post campaign survey was conducted from the 1st to 5th June 2017. Data cleaning was performed from the 1st to 6th of June, analyzed and results shared 3 days later in a workshop (on June 9th 2017) giving the time to implement recommendations before the second-round campaign that took place from June 15th to 21st, 2017. The workshop was organised and implemented in the region where the campaign was implemented with the participation of head of health areas, district medical teams and supervisors, international partners, regional and ministerial supervisors of the campaign. During this workshop, administrative (from campaign teams) results were presented followed by the results of the survey. This included the background, objectives, methods, results and recommendations. Each recommendation was discussed regarding its relevance, feasibility of its implementation in terms of timing and resources and on who has to implement it. From this discussion, the list of recommendations to be implemented before the and during the second-round campaign was done and responsibilities for implementation defined. The timeline of the implementation of activities is presented in Fig. 2.

### Ethical considerations

All heads of households were informed of the survey and their oral consent was obtained before interviewing household members. Adults (21 years and above) were all informed and their written consent was obtained. Assent from children (12-20 years) was obtained and parental written consent was required for children aged less than 12. The privacy of participants was protected by coding personnal data. All the data collected were saved in an online database protected by a password. The ethical approval was obtained from the Cameroon National Ethics Committee for Human Health Research.

## Results

### Characteristics of participants of surveys conducted at the end of the first and second rounds

Out of the 120 clusters expected, 119 (99.1%) and 117 (97.5%) were reached in the first and second rounds respectively. The missed clusters were geographically inaccessible during the survey mainly due to poor road conditions. One thousand two hundred and sixty-five (81.1%) and 1417 of 1560 (90.8%) expected households were included in the first and second rounds respectively. Four (0.2%) and 06 (0.4%) heads of households refused to participate. In the included households, 4372 and 4840 people were eligible and enrolled both for the first and second rounds. The intra cluster correlation in terms of respondents’ vaccination status per cluster varied from 0.02 to 0.05 with a design effect of 2.76. Table 1 shows the distribution of the enrolled population per sub group for each round. The children and gender distribution of the participants in both rounds were very similar. There was no individual refusal.

**Table 1:** Distribution of participants per sub group and per round

		Children*	Female	Male
	**Participants**	**n (%)**	**n (%)**	**n (%)**
Round 1	4372	1701 (38.9)	1479 (33.8)	1192 (27.3)
Round 2	4840	1912 (39.5)	1693 (35.0)	1235 (25.5)

*Children=1-9 years old

### OCV and communication coverage from the survey conducted at the end of the first-round campaign

#### OCV and communication coverage

Table 2 gives the distribution of households (HH) coverage estimation with campaign messages per health area at the end of the first round vaccination campaign. We noted that the district communication coverage was estimated at 92.1%. Among health areas, this coverage seemed relatively heterogeneous, ranging from 78.7% in Sir health area to 98.0% in Rhumsiki health area. Table 2 also presents per health area and per sub group, the vaccination coverage estimated from reports of HH representatives and vaccination coverage estimated from evidence of vaccination (card/Finger mark). The district vaccination coverage was estimated at 97.3% based on recall and at 81.0% based on evidence. The vaccination coverage estimated based on evidence per health area seemed relatively heterogeneous ranging from 60.2% in Sir Health area to 97.5% in Rhumzou health area. The lowest vaccination coverage estimated based on evidence was observed in Sir Health area where the communication coverage was also the lowest.

Concerning the distribution of vaccination coverage per sub group estimated from evidence. It ranged from 75.0% in the male sub group to 85.6% in children sub group.

The probability of being vaccinated with vaccination card or finger mark when the participant declared so after the first round in the health district of Mogode was 83.2%.

**Table 2:** Distribution of communication and vaccination coverage per health area and sub group during the first round of the Mogode 2017 OCV campaign

	Communication coverage (*N* = 1263)	Vaccination coverage estimated from recall (*N* = 4372)	Vaccination coverage estimated from evidence (*N* = 4372)
	n (%[95% Confidence Intervals (CI)])	n (%[CI])	n (%[CI])
**Health areas**			
Houpou	129 (97.0 [96.2, 97.7])	400 (91.7 [96.8, 97.3])	349 (84.7 [84.1, 85.3])
Kila	113 (89.7 [88.3, 91.0])	297 (98.7 [98.5, 98.9])	247 (82.1 [81.4, 82.7])
Kortchi	198 (90.8 [89.8, 91.7])	532 (95.9 [95.5, 96.2])	455 (82.0 [81.3, 82.6])
Kossehone	127 (96.9 [96.1, 96.7])	443 (98.0 [97.8, 98.2])	319 (70.6 [69.8, 71.3])
Mogode	161 (96.4 [95.7, 97.1])	644 (97.6 [97.3, 97.8])	607 (92.0 [91.6, 92.4])
Rhumsiki	150 (98.0 [97.4, 98.5])	561 (95.9 [95.5, 96.3])	534 (91.3 [90.7, 91.8])
Rhumzou	153 (95.0 [94.1, 95.8])	686 (99.3 [99.1, 99.4])	674 (97.5 [97.3, 97.8])
Sir	137 (78.7 [77.2, 80.2])	693 (96.8 [96.6, 97.0])	431 (60.2 [59.6, 60.8])
**Sub Group**			
Children		1678 (98.7 [98.6, 98.8])	1486 (85.9 [85.6, 86.2])
Female		1428 (96.6 [96.4, 96.8])	1211 (80.4 [80.0, 80.8])
Male		1150 (96.5 [96.4, 96.8])	916 (75.0 [74.5, 75.5])
**District**	**1168 (92.5 [92.1, 92.8])**	**4256 (97.4 [97.3, 97.5])**	**3616 (81.0 [80.8, 81.3])**

#### Reasons of non-vaccination after the first round

Table 3 presents mains reasons why responding campaign population targets were not vaccinated during the first round vaccination. The main reasons included being absent (49.4%) and not been aware (18.2%).

**Table 3:** Main reasons of non-vaccination for the first round survey

Reasons for non-vaccination	Frequency (*N* = 116)	Proportion (%[CI])
Absent	60	51.7 [42.3, 61.1]
Not aware	23	19.8 [13.0, 28.3]
Do not believe in vaccine	4	3.4 [0.9, 8.6]
Fear	2	1.7 [0.2, 6.1]
Vaccinators did not come to household	2	1.7 [0.2, 6.1]

#### Timing of first round survey results dissemination and recommendations from these results

The results of the survey conducted after the first round campaign were presented to campaign organizers five days before the beginning of the second round OCV campaign. This time allowed the implementation of following recommendations that were agreed after discussion by these organizers. These include:

- Each head of health area was charged to:
  - define new itineraries for vaccination and communication teams to ensure coverage of communities with low coverage in the first round,
  - adoption of new vaccination strategies by creating temporary vaccination post in public places to vaccinate targeted population that missed to be vaccinated during door-to-door visits,
- Communication teams had to explain to head of households how important it is to keep vaccination card delivered to each vaccinated person;
- Vaccination team had to ensure finger paint as additional evidence during the second round (as many parents who received vaccination card as evidence of vaccination were absent during the survey).

Needed actions were taken by competent teams to ensure these recommendations were implemented.

### Vaccination coverage from the survey conducted after the second round vaccination campaign

#### Two, single, and zero dose coverage estimated from recall and from evidence

Table 4 presents per health area, the distribution of the two and the single dose vaccination coverage estimated from recall of HH representative and from evidence. The two, and single dose coverage estimated from HH representative recall were 94.0%, and 2.2% respectively. The two, and single dose coverages estimated from evidence were 80.1%, and 1.8% respectively. The two doses coverage estimated from evidence ranged from 67.4% in Sir health area to 96.7% in Rhumzou health area. Concerning the zero dose vaccination coverage, we estimated it at 3.8%. The highest proportion of persons who had not been vaccinated was observed in Mogode health area. Table 4 also reported the situation of vaccination coverage estimated per sub group.

The probability of being vaccinated with vaccination card or finger mark when the participant declared so after the second round in the health district of Mogode was 89.0% for single dose and 85.2% for two doses.

**Table 4:** Distribution of participants according to number of vaccine doses received during the campaign per health area

	Vaccination coverage (N = 4840)	Vaccination coverage estimated from recall (N = 4840)		Vaccination coverage estimated from evidence^a^ (N = 4840)	
	**Zero dose**	**Two dose**	**Single dose**	**Two dose**	**Single dose**
	**n (%[CI])**	**n (%[CI])**	**n (%[CI])**	**n (%[CI])**	**n (%[CI])**
**Health areas**					
Houpou	21 (5.8 [5.5, 6.2])	324 (90.2 [89.8, 90.7])	29 (8.1 [7.6, 8.5])	316 (88.0 [87.7, 88.5])	29 (8.1[7.6, 8.5])
Kila	24 (4.5 [4.1, 4.9])	490 (91.9 [91.4, 92.4])	35 (6.6 [6.1, 7.0])	443 (83.1 [82.5, 83.8])	33 (6.2 [5.8, 6.6])
Kortchi	10 (1.7 [1.5, 1.9])	574 (95.2 [94.8, 95.5])	26 (4.3 [4.0, 4.7])	496 (82.3 [81.6, 82.9])	25 (4.1 [3.8, 4.5])
Kossehone	18 (2.2 [2.0, 2.5])	770 (96.3 [95.9, 96.6])	30 (3.8 [3.4, 4.1])	693 (86.6 [86.0, 87.2])	28 (3.5 [3.2, 3.8])
Mogode	49 (6.3 [6.0, 6.7])	710 (92.0 [91.6, 92.4])	48 (6.2 [5.9, 6.6])	583 (75.5 [74.9, 76.1])	45 (5.8 [5.5, 6.2])
Rhumsiki	26 (5.5 [5.0, 5.9])	449 (94.3 [93.8, 94.8])	20 (4.2 [3.8, 4.6])	313 (65.8 [64.8, 66.7])	15 (3.2 [2.8, 3.5])
Rhumzou	15 (2.3 [2.0, 2.5])	637 (96.7 [96.4, 96.9])	15 (2.3 [2.0, 2.5])	637 (96.7 [96.4, 96.9])	15 (2.3 [2.0, 2.5])
Sir	17 (2.7 [2.5, 2.9])	608 (95.3 [95.0, 95.6])	21 (3.3 [3.1, 3.5])	430 (67.4 [66.8, 68.0])	11 (1.7 [1.6, 1.9])
**Sub group**					
Children	41 (2.3 [2.2, 2.4])	1848 (96.4 [96.2, 96.6])	57 (3.1 [3.0, 3.3])	1619 (83.9 [83.6, 84.2])	52 (2.8 [2.7, 3.0])
Female	76 (4.5 [4.3, 4.7])	1576 (92.9 [92.7, 93.2])	92 (5.6 [5.4, 5.8])	1347 (79.0 [78.6, 79.4])	81 (4.9 [4.7, 5.1])
Male	63 (5.3 [5.1, 5.6])	1138 (91.7 [91.4, 92.0])	75 (6.3 [6.1, 6.6])	945 (75.8 [75.3, 76.3])	68 (5.7 [5.5, 6.0])
**District**	**180 (3.8 [3.7, 3.9])**	**4562 (94.0 [93.8, 94.1])**	**224 (4.8 [4.7, 4.9])**	**3911 (80.1 [79.9, 80.4])**	**201 (4.3 [4.2, 4.4])**

^a^The evidence of vaccination was either vaccination card or finger paint

#### Comparison of single dose immunization coverage between the first and the second-round vaccination campaign

Table 5 compares between the first and the second dose vaccination coverages with oral cholera vaccine. This was done to assess if actions taken from the recommendations of the first-round survey improved the coverage of the second round. It is noted that the coverage was significantly increased in the second round compared to the first round (X^2^=69.0 and p-value**<** 0.00). This coverage was not significantly different in health areas where the first-round coverage was relatively high (Mogode, Rhumsiki). Concerning the coverage per sub group, we noted a statistically significant increase in each sub group between the first and the second round.

**Table 5:** Comparison of vaccination coverage during each campaign round based on the results of the two surveys

	First round coverage from evidence N = 4372		Second round coverage from evidence N = 4840		X² (Pearson)	*P*-value
	**N**	**n (%[CI])**	**N**	**n (%[CI])**		
**Health Area**						
Houpou	412	349 (84.7 [84.1, 85.3])	359	336 (93.6 [93.2, 94.0])	14.4	< 0.00
Kila	301	247 (82.1 [81.4, 82.7])	533	469 (88.0 [87.4, 88.6])	5.1	0.01
Kortchi	555	455 (82.0 [81.3, 82.6])	603	551 (91.4 [90.9, 91.8])	21.6	< 0.00
Kossehone	452	319 (70.6 [69.8, 71.3])	800	725 (90.6 [90.1, 91.1])	82.4	< 0.00
Mogode	660	607 (92.0 [91.6, 92.4])	772	675 (87.4 [86.9, 87.9])	7.3	0.99
Rhumsiki	585	534 (91.3 [90.7, 91.8])	476	369 (77.5 [76.7, 78.4])	38.1	0.99
Rhumzou	691	674 (97.5 [97.3, 97.8])	659	645 (97.9 [97.6, 98.1])	0.1	0.41
Sir	716	431 (60.2 [59.6, 60.8])	638	528 (82.8 [82.3, 83.2])	82.0	< 0.00
**Sub Group**						
Children	1701	1486 (85.9 [85.6, 86.2])	1912	1781 (92.9 [92.7, 93.1])	34.2	< 0.00
Female	1479	1211 (80.4 [80.0, 80.8])	1693	1471 (86.9 [86.5, 87.2])	14.8	< 0.00
Male	1192	916 (75.0 [74.5, 75.5])	1235	1046 (84.4 [84.1, 84.9])	23.6	< 0.00
**District**	**4372**	**3616 (81.0 [80.8, 81.3])**	**4840**	**4298 (88.7 [88.5, 88.8])**	**69.0**	**< 0.00**

#### Recommendations made from results of survey conducted at the end of the second round

- From the second round end campaign survey, the main weakness identified was the high proportion of single-dose vaccinated persons. This was discussed during the second round evaluation meeting and guided decision making to organize a catch up session targeting those with one dose vaccination.

## Discussion

The results of this study are showing that the immunization coverage estimated on the basis of evidence from the survey conducted after the first round immunization campaign significantly increased in the second round in the Mogode health district and in the health areas where the coverage was low in the first round. It shows that the dropout rate in immunization coverage between the first and second rounds of vaccination was low. This can be attributed to the implementation of recommendations from the survey conducted at the end of the first round of the campaign. These recommendations pointed out health areas with low immunization and communication coverage, reasons for non-vaccination, and insufficient documentation of vaccination. The use of the recommendations from the end of the first round of the campaign shows that the proposed organization of the first-round survey makes it possible to generate and use the results and recommendations of the first round survey in time to improve the coverage of the second round of the campaign.

A two round OCV immunization campaign is recommended to significantly reduce cholera transmission in communities [23, 24]. Two dose vaccination is recommended by the manufacturer from clinical trials and as proven from published reviews, there is a significant higher proportion of persons protected from a two dose vaccination compared to single dose [25, 26]. For this reason, OCV campaigns should vaccinate the maximum of the targeted population in the first round and take measures to ensure that the maximum of the population vaccinated in the first round is reached in the second round. To make sure the maximum population are covered in the first and the second round vaccination campaign, the vaccination team has to include in the micro-planning an efficient campaign monitoring plan as recommended by WHO guidelines [27]. In the monitoring system, the important role of surveys has been defined [28]. To the best of our knowledge, no published study has shown the benefit of using the result of a post first round survey to improve where needed the coverage of the second round.

During a two-round vaccination campaign, it is essential to achieve in the first round, a higher coverage than that targeted by the campaign since for various reasons, not everyone vaccinated in the first round is expected to be vaccinated in the second round. This was illustrated in a mass OCV vaccination campaign using the Shanchol vaccine in India which reported a drop out rate of 25% between the two rounds. In the campaign targeted by the present study, the coordination, communication, supervision, vaccination, monitoring and evaluation teams contributed to achieve documented first dose vaccine coverage of 81.0%. Using the result of the first round survey to improve identified campaign weaknesses, the achieved documented single dose and two dose coverages for the second round were 4.3% and 80.1% respectively. We believe that the increase of the vaccination coverage during the second round and the relatively high double doses were due to the fact that the identified weaknesses from the survey were overcome during the implementation of the second round activities. For example, in the Sir health area which had the lowest communication and vaccination coverage from the results of the first round survey, actions were taken to improve communication coverage during the second round that probably resulted in increasing vaccination coverage in that health area. Other health areas benefited from this strategy whereby their coverages were also improved. In contrast to this study, during an OCV campaign implemented in Nigeria, two surveys were conducted at the first and second rounds of the campaign but the two doses vaccination coverage were significantly lower than the coverage of each round. The gap in the Nigeria study could be explained by the fact that the results of the first round survey were not used to improve campaign coverage in the second round [11]. This supports the necessity of not only conducting a survey after the first round but ensuring the use of its results to improve the second-round coverage.

The first-round survey of the present study mapped the distribution of low communication and vaccination coverage, undocumented vaccination and reasons of non-vaccination. Sharing and discussing these results with the campaign coordinating, supervising and implementing teams induced adjustment regarding communication plan, supervision and vaccination strategies, and documentation of vaccination. Adjustment actions taken from first round survey recommendations included: redefining itineraries of communicators, supervisors and vaccinators; integrating supervisor activities; adding nail painting as additional vaccination proof; deciding for new vaccination point in public places; planning for a third-round vaccination to catch up with single doses vaccination from both rounds. Similar actions taken from results of other campaign monitoring methods have shown benefits regarding vaccination campaign quality and coverage [29–32]. The present study did not document the individual benefit of each intervention implemented from the result of the first-round survey but the coverage of the second round was significantly higher than that of the first round. This was consistent with the majority of health areas.

The two-dose OCV coverage at the end of the second round is the indicator of population protection against cholera [4]. The estimated two doses coverage of the present study was 80.1% for people vaccinated with proof and of 93.8% for people vaccinated based on recall. The coverage of a survey conducted after two rounds differ from one campaign context to another but the tendency is that coverages from reactive campaigns are higher and those of preventive campaigns lower than that of the present study [9, 33–35]. The heterogeneity of the coverage is probably related to interventions included in campaigns and the determinants of coverage in the campaign context [36–38]. Studies conducted after a reactive campaign are probably related to better adherence of the population to the campaign intervention [13].

The present study has as particularity to present the value of zero dose and one dose after two round vaccinations. These indicators can be used to map weaknesses and plan corrective actions to make sure the campaign has its maximum impact. For example, if the single dose coverage after two rounds vaccination is very high it may be used to plan a third round. In the current campaign, one dose vaccination coverage was estimated at 4.3% and justified the planning and implementation of the third-round vaccination. Some studies have reported a single dose vaccination with the estimate varying according to the settings, type and strategies of campaign, method and timing of the coverage assessment [39, 40]. These studies did not report any corrective action taken from the results of their survey.

The estimate of zero dose in the current study was 3.8%. This is an important indicator as it may be used to map the distribution of hard-to-reach populations and also for the micro planning of future campaigns [18].

The main challenge in the actual study was planning and implementing the timely data collection, transmission, management, analysis and use of results for timely planning and implementation of corrective actions between campaign rounds. To overcome this challenge, we were inspired by a study that highlighted benefits of using smart phones to improve data collection timeliness and quality [17]. This was completed by onsite data quality control and submission to the server for daily data management and analysis. In addition, all actors involved in the implementation of the campaign participated in the results dissemination process and were prepared to implement the recommendations of the first-round survey.

The estimated direct cost for the two surveys was $45,345 meaning $16.9 per visited household. This includes cost for staff salary =$11,455, supplies (including telephone, office supplies and communication credit) =$4873, field trips for supervision teams =$10,909, perdiem surveyors =$7200, training=$5455 and results dissemination= $5455. The cost would have been higher if the preexisting list of households was not available to be used for the sampling as it may require undertaking households’ listing and selection.

The present study had some limitations. We cannot conclude from the benefit of the innovation that we evaluated because we did not foresee a control group to compare the effects. Similarly, the design we have adopted does not allow us to determine the effect of a certain number of determinants on the difference in coverage between the first and the second round of vaccination. We propose that a future study be conducted overcoming these weaknesses. The sample size was insufficient to estimate the individual vaccination coverage between health areas in the two surveys and that there was not control group to independently assess the effect. Another limitation was that the study did not collect the data to explain the difference between declared and documented OCV vaccination. It could be explained by the fact that the person who answered for each participant could not have access to the vaccination card of the person targeted by the survey, either because the card was lost and in certain cases, not given during vaccination. The vaccination status of a person declared vaccinated when proof of vaccination cannot be presented is yet to be clarified.

## Conclusions

The present study has shown that it is feasible to conduct a community-based survey collecting and transmitting data via internet at the end of each OCV vaccination campaign round. It suggests that this can be used to improve the planning, resources distribution and procedures of the second-round campaign. It also suggests that, using results of first round OCV campaign to correct weaknesses from the first round contribute to significantly improve the coverage of the second round.

We recommend conducting future studies to estimate the benefit of the present approach in a controlled study and to compare the efficiency of this approach with the practical approach of conducting a survey after the second round of vaccination and with other approaches such as rapid convenience monitoring, LQAS (*lot quality assurance sampling*). Further studies should investigate the cost effectiveness and partners’ acceptability of implementing and using results of between round surveys to improve the campaign coverage. Interventions are to be tested to contribute in reducing the gap between documented and declared vaccination coverages.

## Supplementary Information

**Additional file 1.** Questionnaire of the surveys conducted in the study “An innovative approach in monitoring Oral Cholera Vaccination campaign: integration of a between-round survey”.

## Data Availability

The datasets used and/or analyzed during the current study are available from the corresponding author on reasonable request.

## Abbreviations

HF: Health Facility
ICG: International Coordinating Group on Cholera Vaccine
M&E: Monitoring and Evaluation
OCV: Oral cholera Vaccine
ODK: Open Data Kit
