# Supplementary material for: An innovative approach in monitoring oral cholera vaccination campaign: integration of a between-round survey
Source: BMC Public Health. 2022 Feb 5;22:238. doi: 10.1186/s12889-022-12610-5 (PMC8817499; doi:10.1186/s12889-022-12610-5)
Supplement: Supplementary file 1 — Additional file 1. Questionnaire of the surveys conducted in the study “An innovative approach in monitoring Oral Cholera Vaccination campaign: integration of a between-round survey”. [file 12889_2022_12610_MOESM1_ESM.pdf]

MEILLEUR ACCES AUX SOINS DE SANTE/BETTER ACCESS TO HEALTH CARE

Statut M.A.SANTE du 17-04-2006
Declaration N°001078/RDA/JO6/BAPP
contribuable: M090600046814N

Adress : Yaounde, BiyemAssi Lac, face Wisdom Academic Complex
P.O.Box: 33.490 Yaounde Cameroon
Tel: **(+237)** 222 311 647;681 376118; 694 283602
E-mail: cmmassante@gmail.com; Site web: www.masante-cm.org

Date of the survey (DD/MM/YYYY) _________ Code _________ Vaccination **round** **First round [ ]** **Second round[ ]**

Health area________ Village _________ Cluster N°__________ Household N°______

Respondent: [ ] Father [ ] Mother [] If other, specify……verbal consent [ ]Yes [ ] No

GRID FOR DATA COLLECTION ON VACCINATION COVERAGE

		1	2	3	4	5	6	7	8	9	10
**Age in years**											
**Sex**	Female = 0 ; Male = 1										
**First dose taken ?**	No=0 ; Yes=1										
**Second dose taken ?**	No=0 ; Yes=1										
**First and second doses received ?**	No=0 ; Yes=1										
**Vaccination card**	No=0 ; Yes=1										
**Vaccinated with labelling**	No=0 ; Yes=1										
**Reason (s) for non-vaccination** (Write down proposition(s) given in the column on the right)	a- I was not informed on the campaign;<br>b- I was not informed on vaccination schedules;<br>c- I was not informed on vaccination dates/time in my village;<br>d- I was not informed that i was a target;<br>e- I was absent ;<br>f- I am afraid of vaccine;<br>g- I do not believe on the fact that										

Statut M.A.SANTE du 17-04-2006
Declaration N°001078/RDA/JO6/BAPP
contribuable: M090600046814N

Adress : Yaounde, BiyemAssi Lac, face Wisdom Academic Complex
P.O.Box: 33.490 Yaounde Cameroon
Tel: **(+237)** 222 311 647;681 376118; 694 283602
E-mail: cmmassante@gmail.com; Site web: www.masante-cm.org

	vaccine can protect me ;<br>h- I am not sure to be at risk of cholera ;<br>i- I do not need vaccine to be protected ;<br>j- I did not receive a vaccination team ;<br>k- Others (specify)_______										
**Date of vaccination (DD/MM/YY)**	**First round**										
**Date of vaccination (DD/MM/YY)**	**Second round**										
**Do you/or did the person have any health problem?**	No= 0 ; Yes=1										
**Date of the onset of the problem** (DD/MM/YY)											
**If yest which were the symptoms?** (Write down proposition(s) given in the column on the right)	a- Diarrhea ;<br>b- Vomiting ;<br>c- Nausea ;<br>d- Abdominal pain ;<br>e- Stomach gurgling<br>f- Indigestion<br>g- Buccal ulcers<br>h- Dryness of the mouth<br>i- Cough<br>j- Sore throat<br>k- Fever<br>l- Poor/loss appetite<br>m- Dizziness<br>n- Fainting<br>o- excess sweating Rash										

Statut M.A.SANTE du 17-04-2006
Declaration N°001078/RDA/JO6/BAPP
contribuable: M090600046814N

Adress : Yaounde, BiyemAssi Lac, face Wisdom Academic Complex
P.O.Box: 33.490 Yaounde Cameroon
Tel: **(+237)** 222 311 647;681 376118; 694 283602
E-mail: cmmassante@gmail.com; Site web: www.masante-cm.org

	p- Itching<br>q- Weakness<br>r- Headache<br>s- Insomnia<br>t- Joint pain<br>u- Other (specify)										
**How was it treated?**	a- I did nothing<br>b- I consulted a traditional doctor<br>c- I went to the hospital<br>d- I bought drugs in the street<br>e- Other (specify)										
**How do you/or the person feel actually**	1) Resolved 2) Improving but still continuing<br>3) Remains unresolved<br>4)Recovered but with sequelae<br>5) unknown<br>6) he is dead<br>a-										

Name of the surveyor____ **Name of the supervisor** ______________________________________
